# Behavioral and social drivers of COVID-19 vaccination initiation in the US: a longitudinal study March─ October 2021

**DOI:** 10.1007/s10865-024-00487-1

**Published:** 2024-04-08

**Authors:** Neetu Abad, Kimberly E Bonner, Qian Huang, Brittney Baack, Robert Petrin, Dhiman Das, Megan A. Hendrich, Madeline S. Gosz, Zachary Lewis, David J. Lintern, Helen Fisun, Noel T. Brewer

**Affiliations:** 1 Centers for Disease Control and Prevention, Atlanta, GA USA; 2 Department of Health Behavior, Gillings School of Global Public Health, University of North Carolina, Chapel Hill, NC USA; 3 Ipsos US Public Affairs, Washington, DC USA; 4 Lineberger Comprehensive Cancer Center, University of North Carolina, Chapel Hill, NC USA

**Keywords:** COVID-19, Vaccination, Vaccine initiation, Vaccine intent, Vaccine confidence, Behavioral and social drivers, Longitudinal study

## Abstract

Many studies have examined behavioral and social drivers of COVID-19 vaccination initiation, but few have examined these drivers longitudinally. We sought to identify the drivers of COVID-19 vaccination initiation using the Behavioral and Social Drivers of Vaccination (BeSD) Framework. Participants were a nationally-representative sample of 1,563 US adults who had not received a COVID-19 vaccine by baseline. Participants took surveys online at baseline (spring 2021) and follow-up (fall 2021). The surveys assessed variables from BeSD Framework domains (i.e., thinking and feeling, social processes, and practical issues), COVID-19 vaccination initiation, and demographics at baseline and follow-up. Between baseline and follow-up, 65% of respondents reported initiating COVID-19 vaccination. Vaccination intent increased from baseline to follow-up (*p* < .01). Higher vaccine confidence, more positive social norms towards vaccination, and receiving vaccine recommendations at baseline predicted subsequent COVID-19 vaccine initiation (all *p* < .01). Among factors assessed at follow-up, social responsibility and vaccine requirements had the greatest associations with vaccine initiation (all *p* < .01). Baseline vaccine confidence, social norms, and vaccination recommendations were associated with subsequent vaccine initiation, all of which could be useful targets for behavioral interventions. Furthermore, interventions that highlight social responsibility to vaccinate or promote vaccination requirements could also be beneficial.

**Supplementary Information:**

The online version contains supplementary material available at 10.1007/s10865-024-00487-1.

## Introduction

COVID-19 vaccines are safe and effective tools for preventing and mitigating the effects of SARS-CoV-2 infection (Dooling et al., 2021). Despite the widespread availability of these vaccines since spring of 2021 (Hernandez et al., 2022), initiation of the primary series of COVID-19 vaccines failed to reach the national target of 70% adult vaccination by July 4, 2021 (CDC, 2020). Initiation of booster doses has also fallen short of expectations, with less than two-thirds of US adults vaccinated with the primary series reporting booster vaccine initiation by March 2022 (Lu et al., 2023), and even fewer (17%) reporting having received the updated bivalent booster dose (CDC, 2023; Lu et al., 2023). To better design and implement behavioral interventions that promote vaccination, it is important to understand how intentions to vaccinate change over time, how behavioral and social factors and demographics correlate with COVID-19 vaccine initiation, and how early vaccine intentions are associated with subsequent behaviors. While many studies have examined these associations (Alagarsamy et al., 2022; Baack et al., 2021; Beleche T, 2021; Dubov et al., 2021; Hu et al., 2022; Hudson & Montelpare, 2021; Joshi et al., 2021; Kelly et al., 2021; Khubchandani et al., 2021; Kreps et al., 2020; Malik et al., 2020; Masters N, 2022; Wang et al., 2021), few have explored these behavioral and social drivers comprehensively or how they are longitudinally associated with vaccine initiation (Cameron et al., 2023; Gupta et al., 2021; Hahn et al., 2022; Latkin et al., 2022; Nguyen et al., 2022; Rane et al., 2022; Valckx et al., 2022).

In the current study, we sought to identify longitudinal behavioral and social drivers of COVID-19 vaccine initiation using the Behavioral and Social Drivers of Vaccination (BeSD) Framework (WHO, 2022), which is based on the Increasing Vaccination Model (Brewer et al., 2017). The BeSD Framework has four domains —*thinking and feeling*, *social processes*, *motivation*, and *practical issues*—as key drivers of vaccine initiation. The *thinking and feeling* domain includes disease risk appraisals and vaccine confidence (e.g., beliefs that vaccines are important and safe) (Brewer et al., 2007). The *social processes* domain includes social norms regarding vaccination and recommendations from others to get vaccinated (Brewer, 2021). The *motivation* domain includes vaccine intentions and hesitancy. The *practical issues* domain includes the experience of seeking vaccination, including access barriers and costs accrued (Balasuriya et al., 2021).

We examined behavioral and social drivers of COVID-19 vaccination at baseline (March – April 2021), which was during the phased COVID-19 vaccination eligibility period and weeks before all US adults became eligible for COVID-19 vaccinations (Diesel et al., 2021; Stolberg, 2021), and at follow-up (September – October 2021), which was after five months of COVID-19 vaccination eligibility for all US adults. This study had three specific aims: (1) to assess the proportion of respondents who initiated COVID-19 vaccination by demographic group between baseline and follow-up; (2) to identify baseline behavioral and social drivers that predict COVID-19 vaccine initiation at follow-up; and (3) to assess whether COVID-19 vaccination intent and initiation changed between baseline and follow-up.

## Methods

### Participants

We recruited participants from the Ipsos KnowledgePanel, an online research panel designed to represent the US adult population (Ipsos, 2023). Ipsos KnowledgePanel used address-based probability sampling via the United States Postal Service’s Delivery Sequence File to achieve a national survey panel representative of the US adult population by age, gender, race and ethnicity, education, income, home ownership status, metropolitan area, and Census region. To attain sufficient representation in this study, we oversampled Non-Hispanic Black persons and American Indian or Alaskan Native persons. At baseline, we sampled 5,768 non-institutionalized US adults ages 18 years and older from the panel for the baseline survey, of whom 3,621 (63%) completed the survey. After removing 59 respondents during data cleaning, 3,562 respondents remained in the baseline analytic dataset, among whom 1,997 had not yet initiated the primary series of COVID-19 vaccination.

At follow-up, we invited only participants who were unvaccinated at baseline to participate given our focus on the longitudinal drivers of COVID-19 vaccination. We invited the 1,876 respondents who were unvaccinated at baseline and were still active panel members to take the follow-up survey. Among the 1,876 unvaccinated baseline respondents invited to the follow-up survey, 1,586 completed the survey for an 84.5% follow-up study completion rate. Data cleaning removed respondents based on five quality checks: (1) age differed from profile age by two or more years (*n* = 23 baseline, *n* = 9 follow-up); (2) sex did not match profile sex (*n* = 14 baseline, *n* = 7 follow-up); (3) completed survey in less than 25% of the median survey duration (*n* = 5 baseline, *n* = 6 follow-up); (4) skipped 50% or more of eligible questions (*n* = 23 baseline, *n* = 3 follow-up); or (5) did not answer the two questions on COVID-19 vaccine initiation and vaccine intent (*n* = 3 follow-up). Six respondents failed multiple quality checks at baseline, and five failed more than one check at follow-up. Using the established practices for probability-based panels (Callegaro & Disogra, 2008), the Study-Specific Average Panel Recruitment Rate (RECR), the number of people who responded to the panel recruitment invitation among all those invited to join the panel, was 11.2% among the respondents participating in the baseline survey. The Study-Specific Average Household Profile Rate (PROR), the number of individuals who completed the initial profile survey among all those who were recruited to the panel was 60.6%. The cumulative response rate is calculated as RECR multiplied by PROR multiplied by the study completion rate (62.8%) to obtain a cumulative response rate of 4.3% at baseline. We report follow-up survey completion rates by race/ethnicity and age group (Table S1).

### Procedures

**Fig. 1:**
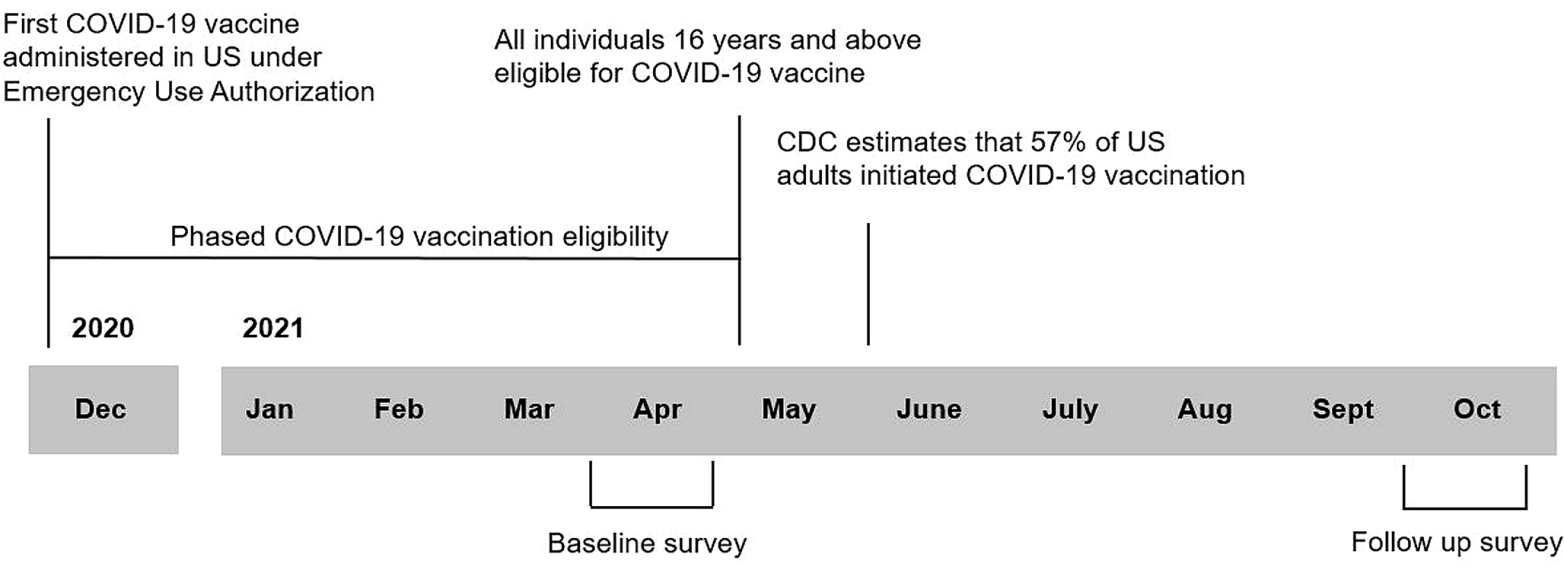
COVID-19 vaccine eligibility and survey administration timeline: Dec 2020—Oct 2021. Source for dates of COVID-19 vaccine first administered in US under an Emergency Use Authorization, all individuals age 16 years and above were eligible for COVID-19 vaccine, and CDC estimate that 57% of US population had initiated vaccination was Diesel, J., Sterrett, N., Dasgupta, S., Kriss, J. L., Barry, V., Vanden Esschert, K., Whiteman, A., Cadwell, B. L., Weller, D., Qualters, J. R., Harris, L., Bhatt, A., Williams, C., Fox, L. M., Meaney Delman, D., Black, C. L., & Barbour, K. E. (2021). COVID-19 Vaccination Coverage Among Adults - United States, December 14, 2020-May 22, 2021. *MMWR Morb Mortal Wkly Rep, 70*(25), 922–927 10.15585/mmwr.mm7025e1

Sampled panel members received email invitations to participate in the baseline survey between March 18–April 2, 2021, and in the follow-up survey between September 16–October 12, 2021 (Fig. 1). Participants completed the online surveys in English (baseline: 96.4%, follow-up: 96.5%) or Spanish (baseline: 3.6%, follow-up: 3.5%). A linguist translated the survey from English to Spanish, and a second linguist reviewed the translation and checked again for accuracy once the Spanish survey was uploaded to the online platform. Survey participants received a small incentive in the form of redeemable points and were entered into a sweepstakes upon survey completion. To increase cooperation, non-responders were offered an additional incentive of $2 or $5 partway through survey fielding. The activity was reviewed by the Centers for Disease Control and Prevention (CDC) and was conducted in a manner consistent with applicable law and CDC policy.^1^

### Measures

#### Demographics

Ipsos KnowledgePanel provided information on participant age (continuous), gender (male, female), race/ethnicity (non-Hispanic White, non-Hispanic Black, Hispanic, non-Hispanic other race or more than one race), education (no high school diploma and no General Educational Development (GED), high school graduate or GED, some college or associate degree, bachelor’s degree or higher), political leaning (extremely liberal, liberal, slightly liberal, moderate, slightly conservative, conservative, extremely conservative), and reported difficulty running errands due to a physical, mental, or emotional condition (yes, no). Residential location was categorized by Census region (Northeast, Midwest, South, West) and rurality (urban, suburban, rural). In addition, the survey included questions on estimated yearly household income in US dollars (in 21 categories, ranging from less than $5,000 to $250,000 or more), being a frontline or essential worker (yes or no/unsure), and mental health status (fair/poor or excellent/very good/good).

#### Behavioral and social drivers

The behavioral and social drivers of vaccination variables were scaled by domain and their component constructs, using a sum of scores on the component survey items. Each of the BeSD constructs had previously been identified and items had undergone cognitive interviewing (WHO, 2022). We also conducted additional pretesting including cognitive interviewing for items added to this survey at baseline. Survey questions, response options, and numeric coding for the summative scale appear in Appendix A. The interpretation of these summative scales varied by construct. For example, a higher score on vaccine confidence indicated higher confidence in COVID-19 vaccines, and a higher score on access indicated easier access and higher satisfaction with the rollout and communications from health authorities. Internal consistency was assessed by correlation for constructs with two variables and Cronbach’s alpha for constructs with more than two variables.

From the *thinking and feeling* domain, the baseline survey assessed the constructs of risk perception and vaccine confidence. Risk perception (α = 0.89) included two questions on concern about getting COVID-19 and fear of getting COVID-19. Vaccine confidence (α = 0.93) was assessed by 11 questions on the importance of COVID-19 vaccines to protect oneself and one’s community against COVID-19, the extent to which respondents felt/anticipated feeling relieved after getting vaccinated, agreement that the COVID-19 vaccination process was likely to be/was unpleasant, perceived safety of COVID-19 vaccines, concern about COVID-19 vaccines causing a serious reaction, agreement that COVID-19 vaccine side effects were likely to be/were bad, worry about long-term side effects from COVID-19 vaccines, perception that vaccine licensure was rushed, and trust in health care providers and agencies that recommended COVID-19 vaccines.

From the social processes domain, the baseline survey assessed the constructs of social norms, vaccine recommendations, and exposure to negative COVID-19 vaccine information. The social norms (α = 0.79) construct included two questions on descriptive social norms (whether family/friends or people at work/school want to get vaccinated) and one question on injunctive norms (whether family/friends want me to get vaccinated). The recommendations construct (α = 0.74) was generated as the sum of five questions on receiving a COVID-19 vaccine recommendation from their personal health care provider, another health care provider, employer, close family, and friends. Exposure to negative COVID-19 vaccine information included two questions (α = 0.84) on seeing or hearing any negative information about the safety/efficacy or other aspects of COVID-19 vaccines.

From the *practical issues* domain, the baseline survey assessed the construct of experience accessing COVID-19 vaccines (α = 0.80). The construct was composed of one question on the difficulty of obtaining a COVID-19 vaccine, three questions on satisfaction with communications from health officials regarding vaccine eligibility, locations, and appointment scheduling, and one question on overall satisfaction with the vaccine rollout.

The follow-up survey assessed: receiving a vaccine recommendation since baseline, exposure to media messaging, social responsibility to receive a vaccine, vaccination requirements, vaccination incentives, and experience with COVID-19 infection. The construct on vaccine recommendations in the last six months was coded to reflect: (1) never received a recommendation or previously received a recommendation at baseline (regardless of follow-up); and (2) received a recommendation at follow-up only. Exposure to media messages promoting or opposing vaccination was recoded as a three-category variable (saw no pro-vaccination message, saw only pro-vaccination messages, or saw both pro and anti-vaccination messages). Social responsibility to receive a COVID-19 vaccine was assessed by one question, categorized as strongly/very strongly agree or somewhat/do not agree with the social responsibility to be vaccinated against COVID-19. Vaccination requirements were assessed using a variable recoded to have two categories (required to get a vaccine for either work or school or not required to get a vaccine for work or school). Vaccination incentives were assessed using a recoded variable with two categories (offered any incentives for getting a COVID-19 vaccine or not offered any incentives for getting a COVID-19 vaccine). Experiences with COVID-19 infection were assessed with two variables including having had COVID-19 in the last six months (categorized as probably/definitely vs. not sure/probably not/definitely not) and knowing someone who became seriously ill or died of COVID-19 (yes or no).

#### Outcomes

The survey assessed two outcomes. The vaccine initiation outcome variable enabled an assessment of whether or not a participant initiated COVID-19 vaccination between the baseline and follow-up surveys. For the second outcome variable, we combined vaccination intention and behavior into a single outcome variable that measured vaccination intent and initiation at follow-up, similar to the stage of change in the transtheoretical model (Prochaska & DiClemente, 1983). The first outcome of interest, vaccine initiation, was assessed with the question, “Have you received at least one dose of a COVID-19 vaccine?” (yes or no). The second outcome of interest, vaccination intent and initiation, was assessed with two questions: the vaccine initiation question (outcome 1) and then for those who had not received at least one dose of a COVID-19 vaccine, the question, “How likely are you to get a COVID-19 vaccine?” Intent was rated on a five-point response scale, ranging from definitely will not to definitely will get vaccinated.

### Data analysis

We report unweighted frequencies and weighted percentages. Baseline survey data were weighted to match the US adult population using benchmarks from the US Census Bureau’s 2019 American Community Survey and the US Census Bureau’s 2020 March Supplement of the Current Population Survey. The follow-up survey data were weighted to match the benchmarks of the unvaccinated population from the baseline survey. Weighting variables were gender, age, race/ethnicity, Census region, metropolitan status, education, household income, language proficiency, and Hispanic origin.

To address item nonresponse, we imputed missing values. We generated 15 imputed data sets using random forest multiple imputation, a procedure that replaces individual missing values with values drawn from a set of probable values (van Buuren, 2018). Rubin’s Rules were used to pool results from analyses conducted separately on each of the individual post-imputation data files (Marshall et al., 2009; Schafer & Graham, 2002). This process allowed us to avoid using listwise deletion when analyzing the data (thus retaining the full representation of the original data set), while also properly reflecting variability in imputed values and their impact on subsequent point estimates and inferences (Marshall et al., 2009; Schafer & Graham, 2002). Regression results are reported using odds ratios, and the statistical significance of model parameter estimates were evaluated using two-tailed tests and a critical alpha of 05.

We calculated the count and weighted proportion vaccine intent and initiation of all respondents (Table 1). We reported the count and the proportion of vaccinated respondents within each demographic group and used logistic regression on the imputed data to assess the bivariate relationship between each of the demographic factors and vaccine initiation at follow-up (Table 2).

**Table 1:** COVID-19 vaccination initiation and intent at follow-up, among respondents who were unvaccinated at baseline (*n =* 1,563)

Vaccine initiation and intent	Unweighted *n*	Weighted %
Definitely will not	237	15.4
Probably will not	135	8.7
Not sure	110	7.3
Probably will	43	3.1
Definitely will	13	0.9
Vaccinated, at least one dose	1,025	64.6
Total	1,563	100.0

**Table 2:** Demographic correlates of COVID-19 vaccine initiation by follow-up, among respondents who were unvaccinated at baseline (*n =* 1,563)

Demographics	Overall		Received ≥ 1 dose of COVID-19 vaccine			Bivariate associations	
	***n***	**%**	***N***	**%**	**95% CI**	***OR***	**95% CI**
**Age (years)** ^**±**^							
18–24	107	12.8	71	66.8	57.6–76.0	Ref	
25–34	288	23.5	174	57.9	51.5–64.3	0.68	0.42–1.12
35–44	323	22.5	216	67.3	61.7–72.9	1.02	0.63–1.67
45–54	281	15.9	187	64.6	58.5–70.7	0.91	0.55–1.48
55–64	371	17.8	257	68.6	63.4–73.9	1.09	0.67–1.76
65–74	147	5.5	92	63.9	55.5–72.2	0.88	0.51–1.52
75+	46	1.9	28	63.6	48.9–78.4	0.87	0.41–1.86
**Gender**							
Male	802	49.7	544	66.7	62.9–70.5	Ref	
Female	761	50.3	481	62.4	58.5–66.4	0.83	0.65–1.05
**Race/ethnicity**							
NH White	962	60.3	615	61.4	58.0-64.7	Ref	
NH Black	258	13.2	162	59.5	52.5–66.4	0.92	0.67–1.27
Hispanic	187	17.8	128	71.7	63.8–79.5	1.59*	1.06–2.40
NH Other	156	8.7	120	80.2	72.7–87.7	2.56**	1.56–4.19
**Education** ^**±**^							
No high school diploma or GED	140	14.0	77	57.4	48.2–66.5	Ref	
High school graduate or GED	438	29.9	249	54.9	49.6–60.2	0.90	0.59–1.39
Some college or associate degree	504	31.3	320	64.1	59.4–68.7	1.33	0.87–2.03
Bachelor’s degree or higher	481	24.9	379	80.9	77.1–84.7	3.15**	2.02–4.93
**Household income** ^**±**^							
Less than $25,000	251	15.9	141	52.9	45.7–60.1	Ref	
$25,000-$49,999	287	18.5	163	56.6	49.9–63.3	1.17	0.78–1.75
$50,000-$74,999	238	16.2	151	64.7	57.8–71.5	1.60*	1.04–2.45
$75,000-$99,999	233	14.7	147	59.7	52.6–66.8	1.29	0.85–1.96
$100,000-$199,999	399	26.8	303	74.3	69.2–79.3	2.45**	1.65–3.62
$200,000 and above	111	7.9	97	86.3	79.1–93.4	5.39**	2.76–10.54
**Political leaning** ^**±**^							
Extremely liberal	58	3.9	50	89.2	80.5–97.9	Ref	
Liberal	197	13.3	171	86.5	80.6–92.4	0.78	0.27–2.27
Slightly liberal	137	9.9	109	78.1	69.6–86.7	0.44	0.15–1.27
Moderate	528	36.6	349	64.5	59.8–69.2	0.22**	0.08–0.57
Slightly conservative	168	10.7	115	66.7	58.4–75.0	0.24**	0.09–0.66
Conservative	307	18.8	149	47.0	40.9–53.1	0.11**	0.04–0.28
Extremely conservative	107	6.9	42	35.9	25.9–45.8	0.07**	0.02–0.19
**Essential or frontline worker status**							
No/Not sure	1142	73.2	768	66.4	63.3–69.6	Ref	
Yes	421	26.8	257	59.4	54.2–64.7	0.74*	0.57–0.96
**Difficulty running errands**							
No	1373	92.9	902	64.5	61.6–67.4	Ref	
Yes	109	7.1	70	63.8	53.6–73.9	0.96	0.60–1.51
**Mental health status**							
Fair/poor	181	12.3	116	59.3	51.2–67.4	Ref	
Excellent/very good/good	1372	87.7	904	65.5	62.6–68.4	1.29	0.90–1.85
**Rurality**							
Urban	513	32.3	351	69.2	64.6–73.7	Ref	
Rural	275	17.7	149	51.7	45.1–58.3	0.48**	0.34–0.67
Suburban	775	50.0	525	66.2	62.3–70.1	0.87	0.66–1.15
**Census region**							
Northeast	286	17.7	215	74.6	68.9–80.3	Ref	
Midwest	315	20.8	205	62.9	56.9–68.9	0.58**	0.39–0.86
South	604	38.2	362	57.3	52.7–61.9	0.46**	0.32–0.65
West	358	23.2	243	70.3	65.0-75.7	0.81	0.54–1.20

*Note* OR = odds ratio. NH = Non-Hispanic. GED = General Educational Development. Ref = reference group. Table reports unweighted frequencies, weighted proportions, and weighted ORs. OR point estimates and confidence intervals are based on 15 multiple imputations. ^±^Treated as continuous variables in logistic regression models.

*p < .05, ** *p < .01*

To identify the behavioral and social drivers of COVID-19 vaccine initiation at follow-up, we assessed the relationship between each construct and vaccine initiation, using a pair of logistic regression models (Table 3). A first model (Model 1) examined behavioral and social drivers at baseline as predictors of COVID-19 vaccine initiation at follow-up. A second model (Model 2) examined the relationship between behavioral and social drivers from both baseline and follow-up and COVID-19 vaccine initiation at follow-up. Model 1 included constructs from the BeSD Framework measured at baseline. Model 2 included all variables in Model 1 as well as follow-up measures on media exposure, social responsibility, vaccine requirements, vaccine incentives, experiences with COVID-19 infection, and vaccine recommendations since baseline. Both models controlled for age, gender, race/ethnicity, education, household income, rurality, US Census region, political ideology, errand difficulty, frontline worker status, and mental health status. Table S2 reports the unadjusted (i.e., not controlling for demographics) relationship between behavioral and social drivers from baseline alone and from baseline and follow-up with COVID-19 vaccine initiation at follow-up. Table S3 reports the bivariate associations between BeSD constructs assessed at baseline and follow-up with COVID-19 vaccine initiation at follow-up.

To assess the association between COVID-19 vaccine intent at baseline and intention and initiation at follow-up, we used the McNemar-Bowker test (Bowker, 1948; McNemar, 1947). Appendix B lists variables included in each analysis. Analyses were conducted using R (Team, 2018).

## Results

### Vaccine initiation

Overall, 64.6% (1,025/1,563) of respondents had initiated COVID-19 vaccination by the time of the follow-up survey (Table 1). Among those who were not vaccinated at follow-up, 2.6% (13/538) said that they definitely would get vaccinated, 8.6% (43/538) said that they probably would get vaccinated, 20.6% (110/538) said that they were not sure about getting vaccinated, 24.7% (135/538) probably would not get vaccinated, and 43.6% (237/538) said that they definitely would not get vaccinated.

COVID-19 vaccine initiation surpassed 70% among the following: respondents in the Northeast (74.6%) and in the West (70.3%); respondents classifying themselves as slightly liberal (78.1%), liberal (86.5%), and extremely liberal (89.2%); respondents with a bachelor’s degree or higher (80.9%); respondents identifying as Hispanic (71.7%) and multi-racial or other non-Hispanic (80.2%); and respondents reporting a household income between $100,000-199,999 (74.3%) and over $200,000 (86.3%) (Table 2). COVID-19 vaccine initiation was less than 55% among the following: respondents with a high school education (54.9%); respondents who reported being conservative (47.0%) or extremely conservative (35.9%); respondents with a household income less than $25,000 (52.9%); and respondents living in rural areas (51.7%) (Table 2).

### Drivers of vaccine initiation at follow-up

#### Thinking and feeling

Higher baseline vaccine confidence was associated with vaccine initiation at follow-up (*OR* = 1.13, *p* < .01). Baseline risk perception was not associated with vaccine initiation in Model 2 (*p* > .05), though Model 1 (baseline and demographic measures only) found that higher risk perception was associated with initiation (*OR* = 1.22, *p* < .01).

#### Social processes

Higher social norms (*OR* = 1.13, *p* < .01) and any vaccine recommendation at baseline (*OR* = 1.12, *p* < .01) were associated with vaccine initiation at follow-up. Exposure to negative information at baseline was not associated with vaccine initiation (*p* > .05). Among constructs assessed at follow-up, agreement to having a social responsibility to receive a COVID-19 vaccine was associated with vaccine initiation (*OR* = 10.22, *p* < .01), as was receiving a vaccine recommendation at follow-up (*OR* = 1.72, *p* < .05). Exposure to vaccination messages (anti- and pro-) at follow-up was not associated with vaccine initiation (*p* > .05).

#### Practical issues

Higher levels of COVID-19 vaccine access experience at baseline were associated with not initiating vaccination (*OR* = 0.89, *p* < .01). Among factors assessed at follow-up, being subject to a vaccination requirement was associated with vaccine initiation (*OR* = 8.71, *p* < .01), while being offered a vaccination incentive was associated not initiating vaccination (*OR* = 0.59, *p* < .05). However, neither having had COVID-19 in the last six months prior to follow-up survey nor knowing someone who became seriously ill or died of COVID-19 were associated with vaccine initiation (*p* < .05).

### Vaccine initiation by baseline intent

**Fig. 2:**
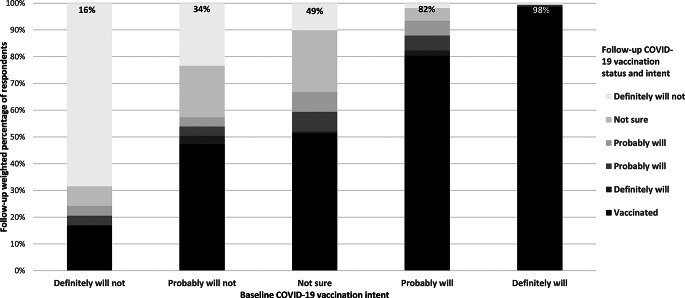
Change in COVID-19 vaccination intent and initiation between baseline (March–April 2021) and follow-up (September–October 2021), among respondents unvaccinated at baseline (*n* = 1,563)

Vaccination intent increased between baseline and follow-up (McNemar-Bowker χ^2^(10) = 447.64, *p* < .001) (Fig. 2). Among those who reported that they definitely would get vaccinated at baseline, 98.4% reported that they had initiated vaccination (563/573). Furthermore, 82.3% (226/271) of those who indicated that they probably would get vaccinated at baseline reported that they had initiated vaccination at follow-up, along with 49.2% (114/229) of those who indicated they weren’t sure at baseline, 34.0% (81/223) of those who indicated they probably would not vaccinate at baseline, and 15.6% (41/267) of those who indicated they definitely would not vaccinate at baseline.

## Discussion

Behavioral and social drivers were important predictors of COVID-19 vaccine initiation among a nationally representative sample of unvaccinated US adults. Almost two thirds had initiated COVID-19 vaccination within six months, and most people who intended to vaccinate at baseline subsequently initiated vaccination by follow-up. However, two-thirds of those who indicated that they probably would not vaccinate and 84% of those who indicated that they definitely would not get vaccinated remained unvaccinated at follow-up. This speaks to the difficulty of convincing those entrenched in their opposition to vaccination to be vaccinated (Beleche T, 2021). Interventions that foster social norms favoring vaccination, make vaccines easier to access, or otherwise reach populations who intend to vaccinate or are open to vaccination may be promising (Omari et al., 2023; Shen et al., 2023).

### Behavioral and social drivers of COVID-19 vaccine initiation at follow-up

#### Thinking and feeling

Respondents who had higher confidence in COVID-19 vaccines at baseline were more likely to initiate vaccination at follow-up than those reporting lower vaccine confidence at baseline. The association between baseline vaccine confidence and COVID-19 vaccine initiation at follow-up remained relatively stable when adjusted for demographics and other factors assessed at follow-up, indicating its central role in affecting vaccination behavior. As seen elsewhere (Alagarsamy et al., 2022; Wang et al., 2021), our study found higher perceived risk of COVID-19 at baseline predicted vaccine initiation, but this association was mitigated when other factors assessed at follow-up were in the model (Table 3) (Rane et al., 2022). Thus, delivering interventions that focus solely on increasing risk perception of contracting COVID-19 are unlikely to meaningfully improve vaccine initiation in the US. An alternative interpretation is that these added variables mediate the association of risk perception and vaccine initiation.

**Table 3:** Association of behavioral and social drivers of vaccination with COVID-19 vaccine initiation by follow-up, among respondents who were unvaccinated at baseline, adjusted for demographics

Construct	Assessment	Unvaccinated Respondents Mean (SD)	Vaccinated Respondents Mean (SD)	Model 1 Baseline		Model 2 Baseline & follow-up			
				OR	95% CI	OR		95% CI	
**Thinking and feeling**									
Vaccine confidence	Baseline	21.70 (6.79)	33.32 (7.49)	1.15**	1.12–1.19	1.13**		1.09–1.16	
Risk perception	Baseline	3.70 (1.81)	5.14 (1.78)	1.22**	1.09–1.35	1.08	0.95–1.22		
**Social processes**									
Social norms	Baseline	7.31 (2.71)	11.33 (2.83)	1.16**	1.08–1.25	1.13**		1.05–1.22	
Exposure to negative information	Baseline	5.01 (1.42)	4.39 (1.69)	0.96	0.85–1.08	1.00		0.87–1.15	
Recommendation	Baseline	6.76 (2.38)	9.16 (3.22)	1.11**	1.03–1.18	1.12**		1.03–1.22	
Recommendation at follow-up	Follow-up	0.21 (0.41)	0.17 (0.37)	-	-	1.72*		1.06–2.81	
Social responsibility to vaccinate	Follow-up	0.08 (0.27)	0.71 (0.45)	-	-	10.22**		6.11–17.07	
Saw only pro-vaccination messages	Follow-up	0.57 (0.50)	0.58 (0.49)	-	-	0.66		0.38–1.13	
Saw both pro- and anti-vaccination messages	Follow-up	0.25 (0.43)	0.26 (0.44)	-	-	0.88		0.48–1.62	
**Practical issues**									
Access experience	Baseline	12.37 (2.97)	12.73 (3.35)	0.91**	0.86–0.96	0.89**		0.84–0.94	
Vaccine requirement	Follow-up	0.03 (0.17)	0.21 (0.41)	-	-	8.71**		3.39–22.37	
Vaccine incentives	Follow-up	0.22 (0.42)	0.16 (0.37)	-	-	0.59*		0.36–0.95	
Had COVID-19 in the last 6 months	Follow-up	0.17 (0.38)	0.07 (0.25)	-	-	0.57		0.30–1.07	
Know someone who became seriously ill or died of COVID-19	Follow-up	0.42 (0.49)	0.48 (0.50)	-	-	0.75		0.51–1.09	

*Note* OR = Odds ratio. SD = standard deviation. Means, SDs, ORs point, and confidence intervals are based on 15 multiple imputations. Models adjusted for demographics: age, gender, race/ethnicity, education, household income, rurality, Census region, political ideology, errand difficulty, frontline worker status, and mental health. Model 2 assessed recommendations to vaccinate during both the baseline and follow-up survey.

*p < .05, **p < .01

#### Social processes

Those who received recommendations to get vaccinated and those experiencing positive social norms to vaccinate at baseline were more likely to initiate vaccination than those who did not report these social processes. The associations between recommendations and COVID-19 vaccine initiation across time and by family, friends, and healthcare providers underscore the importance of recommendations in promoting vaccine initiation and have been reported in a recent cross-sectional assessment (Bonner et al., 2023). The strong association between social responsibility and vaccine initiation suggests that the motivational influence of social responsibility could be leveraged to encourage vaccine initiation, as demonstrated elsewhere (Bohm et al., 2019; Huang et al., 2023; Li et al., 2016). While exposure to negative information about vaccines is often cited as an explanation for why some individuals and communities are unvaccinated (Islam et al., 2021), our study did not find an association with vaccine initiation. It is possible that the diminishing attention towards the COVID-19 pandemic over time could have blunted the impact of exposure to negative information (Dyer & Kolic, 2020). This finding highlights the importance of considering how the amount of information available on a vaccine could mediate the relationship between exposure to negative information and vaccine initiation.

#### Practical issues

Those who reported a poorer experience accessing COVID-19 vaccines were more likely to report being vaccinated during the follow-up survey than those who did not indicate these factors at baseline. This surprising finding might signal the greater motivation of those who faced early difficulties getting vaccinated, or it might be attributed to strategies that were implemented to address access barriers between April and September 2021 (Tewarson et al., 2021). Furthermore, the relatively low vaccine initiation rates reported by frontline workers identified in this study and elsewhere (Henneberger et al., 2022; Prince et al., 2022) could be attributed to limited access to COVID-19 vaccines among non-healthcare frontline workers, and could be addressed, in part, by increasing workplace flexibilities to receive a vaccination, as reported in an assessment of non-healthcare frontline workplaces in Chicago (Lendacki et al., 2023). Among the items assessed at follow-up, vaccination requirements had a strong association with vaccine initiation, consistent with another nationally-representative survey (Lee et al., 2022). Given this finding, equitable, clearly communicated vaccine requirement policies could provide an opportunity to increase vaccine initiation (Mello et al., 2020) particularly among health care workers (Emanuel & Skorton, 2021). Consistent with a systematic review assessing the impact of incentives on COVID-19 vaccination (Mardi et al., 2022), this study did not find that incentives were associated with greater likelihood of vaccine initiation. However, evidence suggests that targeted incentives can improve vaccine initiation when they are certain, delivered immediately after vaccination, and desired by recipients (Brewer et al., 2022).

### Strengths and limitations

This study has several strengths and limitations. Strengths include the collection of comprehensive behavioral and social drivers, using survey items previously validated via cognitive interviewing (WHO, 2022), a rarity among measures of vaccination confidence (Shapiro et al., 2021); the use of a nationally-representative sample; and the longitudinal nature of the study. Limitations include the exclusion of institutionalized individuals or those without internet access, although the panel attempted to include the latter group by providing internet-enabled devices to respondents who need it during recruitment. Second, we anticipate potential selection bias amongst KnowledgePanel respondents, which we addressed by weighting responses to the non-institutionalized US adult population and by gender, age, race/ethnicity, Census region, metropolitan status, education, household income, language proficiency, and Hispanic origin. Third, the cooperation rate ranged from 63% of those invited to the baseline survey to 85% of those invited to complete the follow-up survey, although these rates are consistent with other nationally-representative surveys. Fourth, we could not infer causality, and whether these associations would generalize to other countries or time points remains to be established. Thus, we restrict the interpretation of these findings to the pandemic time period and US population in which they were collected.

## Conclusions

Most US adults who were unvaccinated in spring 2021 initiated COVID-19 vaccination within six months of widespread vaccine availability. Baseline confidence in COVID-19 vaccines, social norms promoting vaccination, and recommendations from any source to vaccinate all predicted vaccine initiation during follow-up. In addition, higher social responsibility to vaccinate and vaccination requirements were also associated with vaccine initiation. These key behavioral and social drivers can be leveraged for future interventions, which could increase vaccine initiation for both the primary series, booster doses, and now annual doses of COVID-19 vaccines. More broadly, these understandings may be relevant to future vaccine-preventable disease outbreaks, enabling a rapid deployment of interventions that address key drivers of vaccination.

### Electronic supplementary material

Below is the link to the electronic supplementary material.

Supplementary Material 1

## Data Availability

Available upon request to authors.

## Footnotes

1 § See e.g., 45 C.F.R. part 46, 21 C.F.R. part 56; 42 U.S.C. § 241(d); 5 U.S.C. § 552a; 44 U.S.C. § 3501 et seq.

## Code availability

Available upon request to authors.
