# Supplementary material for: Behavioral and social drivers of COVID-19 vaccination initiation in the US: a longitudinal study March─ October 2021
Source: J Behav Med. 2024 Apr 8;47(3):422–33. doi: 10.1007/s10865-024-00487-1 (PMC11026250; doi:10.1007/s10865-024-00487-1)
Supplement: Supplementary file 1 — Supplementary Material 1 [file 10865_2024_487_MOESM1_ESM.docx]

**Table S1.** Follow-up survey completion by demographic groups.

|  |  | Baseline survey  Completed | Follow-up  Total Assigned | Follow-up  Total Cleaned Complete | Follow-up  % Completion  from Assigned | Follow-up  % Completion from baseline survey |
| --- | --- | --- | --- | --- | --- | --- |
| Gender | Male | 996 | 950 | 802 | 84.4 | 80.5 |
|  | Female | 1001 | 926 | 761 | 82.2 | 76.0 |
| Age | 18–29 years | 362 | 335 | 257 | 76.7 | 71.0 |
|  | 30–49 years | 774 | 723 | 586 | 81.1 | 75.7 |
|  | 50–64 years | 631 | 596 | 527 | 88.4 | 83.5 |
|  | 65 + years | 230 | 222 | 193 | 86.9 | 83.9 |
| Race | White, non-Hispanic | 1203 | 1119 | 962 | 86.0 | 80.0 |
|  | Black, non-Hispanic | 320 | 311 | 258 | 83.0 | 80.6 |
|  | Other, non-Hispanic | 139 | 133 | 106 | 79.7 | 76.3 |
|  | Hispanic | 270 | 252 | 187 | 74.2 | 69.3 |
|  | 2 + races, non-Hispanic | 65 | 61 | 50 | 82.0 | 76.9 |
| Other demographic groups | Non-metro area | 272 | 252 | 198 | 78.6 | 72.8 |
|  | Less than high school | 187 | 171 | 140 | 81.9 | 74.9 |
| Overall |  | 1997 | 1876 | 1563 | 83.3 | 78.3 |

* Excludes those who did not answer gender or age questions in the survey.

**Table S2**. Association of behavioral and social drivers of vaccination with COVID-19 vaccine initiation by follow-up, among respondents who were unvaccinated at baseline, unadjusted for demographics.

| **Construct** | **Assessment** | **Model 1**  **Baseline** | | **Model 2**  **Baseline & follow-up** | |
| --- | --- | --- | --- | --- | --- |
|  |  | *OR* | 95% CI | *OR* | 95% CI |
| **Thinking and feeling** |  |  |  |  |  |
| Vaccine confidence | Baseline | 1.15** | 1.12–1.18 | 1.13** | 1.09–1.16 |
| Risk perception | Baseline | 1.15** | 1.05–1.27 | 1.00 | 0.89–1.13 |
|  |  |  |  |  |  |
| **Social processes** |  |  |  |  |  |
| Social norms | Baseline | 1.19** | 1.11–1.28 | 1.17** | 1.09–1.26 |
| Exposure to negative information | Baseline | 1.00 | 0.89–1.11 | 1.03 | 0.90–1.18 |
| Recommendation | Baseline | 1.12** | 1.05–1.19 | 1.11* | 1.02–1.20 |
| Recommendation at follow-up | Follow-up | - | - | 1.57 | 0.97–2.54 |
| Social responsibility to vaccinate | Follow-up | - | - | 8.64** | 5.29–14.14 |
| Saw only pro-vaccination messages | Follow-up | - | - | 0.95 | 0.56–1.61 |
| Saw both pro- and anti-vaccination messages | Follow-up | - | - | 1.11 | 0.60–2.04 |
|  |  |  |  |  |  |
| **Practical issues** |  |  |  |  |  |
| Access experience | Baseline | 0.92** | 0.88–0.97 | 0.90** | 0.85–0.95 |
| Vaccine requirement | Follow-up | - | - | 9.61** | 3.79–24.35 |
| Vaccine incentives | Follow-up | - | - | 0.67 | 0.43–1.04 |
| Had COVID-19 in the last 6 months | Follow-up | - | - | 0.62 | 0.34–1.12 |
| Know someone who became seriously ill or died of COVID-19 | Follow-up | - | - | 0.81 | 0.56–1.17 |

*Note.* OR = Odds ratio. SD = standard deviation. Means, SDs, ORs point, and confidence intervals are based on 15 multiple imputations. Model 2 assessed recommendations to vaccinate during both the baseline and follow-up survey.

**p* < 0.05, ***p* < 0.01

**Table S3**. Bivariate associations between behavioral and social drivers of vaccination and COVID-19 vaccine initiation by follow-up, among respondents who were unvaccinated at baseline.

| **Construct** | **Time** | **Bivariate Association** | |
| --- | --- | --- | --- |
|  |  | *OR* | *95% CI* |
| **Thinking and feeling** |  |  |  |
| Vaccine confidence | Baseline | 1.21** | 1.19–1.24 |
| Risk perception | Baseline | 1.57** | 1.44–1.70 |
|  |  |  |  |
| **Social processes** |  |  |  |
| Social norms | Baseline | 1.57** | 1.48–1.65 |
| Exposure to negative information | Baseline | 0.78** | 0.72–0.84 |
| Recommendation | Baseline | 1.34** | 1.28–1.42 |
| Recommendation at follow-up | Follow-up | 0.73* | 0.54–0.99 |
| Social responsibility to vaccinate | Follow-up | 28.76** | 18.59–44.50 |
| Saw only pro-vaccination messages | Follow-up | 1.21 | 0.86–1.71 |
| Saw both pro- and anti-vaccination messages | Follow-up | 1.22 | 0.84–1.79 |
|  |  |  |  |
| **Practical issues** |  |  |  |
| Access experience | Baseline | 1.04* | 1.00-1.07 |
| Vaccine requirement | Follow-up | 9.58** | 5.34–17.16 |
| Vaccine incentives | Follow-up | 0.66** | 0.49–0.89 |
| Had COVID-19 in the last 6 months | Follow-up | 0.35** | 0.24–0.51 |
| Know someone who became seriously ill or died of COVID-19 | Follow-up | 1.26 | 0.99–1.60 |

*Note.* OR point estimates and confidence intervals are based on 15 multiple imputations. **p* < 0.05. ***p* < 0.01.
